# Bioaccumulation and Toxicity of Cadmium, Copper, Nickel, and Zinc and Their Mixtures to Aquatic Insect Communities

**DOI:** 10.1002/etc.4663

**Published:** 2020-03-26

**Authors:** Christopher A. Mebane, Travis S. Schmidt, Janet L. Miller, Laurie S. Balistrieri

**Affiliations:** ^1^ Idaho Water Science Center US Geological Survey Boise Idaho; ^2^ Colorado Water Science Center US Geological Survey Ft. Collins Colorado; ^3^ Fort Collins Science Center US Geological Survey Ft. Collins Colorado; ^4^ Minerals, Energy, and Geophysics Science Center US Geological Survey, Geology Grafton Wisconsin

**Keywords:** Metal mixture toxicity, Experimental streams, Aquatic insects, Accumulation, Dietary exposure, Biotic ligand models, Species sensitivity distributions

## Abstract

We describe 2 artificial stream experiments that exposed aquatic insect communities to zinc (Zn), copper (Cu), and cadmium (year 2014) and to Zn, Cu, and nickel (year 2015). The testing strategy was to concurrently expose insect communities to single metals and mixtures. Single‐metal tests were repeated to evaluate the reproducibility of the methods and year‐to‐year variability. Metals were strongly accumulated in sediments, periphyton, and insect (caddisfly) tissues, with the highest concentrations occurring in periphyton. Sensitive mayflies declined in metal treatments, and effect concentrations could be predicted effectively from metal concentrations in either periphyton or water. Most responses were similar in the replicated tests, but median effect concentration values for the mayfly *Rhithrogena* sp. varied 20‐fold between the tests, emphasizing the difficulty comparing sensitivities across studies and the value of repeated testing. Relative to the single‐metal responses, the toxicity of the mixtures was either approximately additive or less than additive when calculated as the product of individual responses (response addition). However, even less‐than‐additive relative responses were sometimes greater than responses to similar concentrations tested singly. The ternary mixtures resulted in mayfly declines at concentrations that caused no declines in the concurrent single‐metal tests. When updating species‐sensitivity distributions (SSDs) with these results, the mayfly responses were among the most sensitive 10th percentile of available data for all 4 metals, refuting older literature placing mayflies in the insensitive portion of metal SSDs. Testing translocated aquatic insect communities in 30‐d artificial streams is an efficient approach to generate multiple species effect values under quasi‐natural conditions that are relevant to natural streams. *Environ Toxicol Chem* 2020;39:812–833. Published 2020 Wiley Periodicals, Inc. on behalf of SETAC. This article is a US government work, and as such, is in the public domain in the United States of America.

## Graphical abstract

Concepts of periphyton‐mediated pathways of exposure of bioavailable metals to stream insects. (Commissioned artwork by Amy McMahon.)

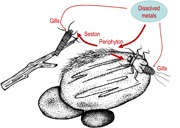

## INTRODUCTION

The present study was motivated by 3 logical disconnects in common risk‐assessment practices for metals in relation to metal mining. First, metals in aquatic environments always occur in mixtures, but risk assessments and environmental quality criteria or standards are commonly developed as if metals occurred one by one in the environment. Second, discharges from metal mining activities often drain to small streams in which the dominant macrofauna are aquatic insects, yet aquatic insects are rare in the aquatic toxicity testing data sets relied on to develop criteria to protect aquatic life. Third, existing toxicity test data for aquatic insects and metals are most commonly from short‐term, water‐only exposures, whereas in the environment, insects would be exposed to metals both through diet and through water (more explanation of these disconnects follows). Our efforts to address these 3 disconnects include a series of experimental stream studies, field surveys, and modeling. In the present study, we report on 2 experiments in which we tested translocated insect communities with cadmium (Cd), copper (Cu), nickel (Ni), and zinc (Zn) singly and in combinations.

Aquatic toxicologists have tested mixtures of metals for decades, mostly in acute combinations that produced conflicting or idiosyncratic conclusions of whether the toxicity of metal mixtures could be considered additive, more than additive (synergistic), or less than additive (antagonistic) relative to the responses of single‐metal effects (Norwood et al. 2003; Vijver et al. 2011). One of the reasons for conflicting reports on additive toxicity was that in many cases comparisons of single‐metal and mixture exposures had been made across tests conducted at different times, and the between‐test variability in responses confounded mixture comparisons (De Laender et al. 2009). More recently, efforts have been made to synthesize metal mixture toxicity within modeling frameworks that account for the bioavailability of metals in water and predict accumulation of metals in tissues and subsequent toxicity. Important limitations of many synthesis approaches include the artificiality of many experimental approaches that may, for example, address acute responses using cocktail metal combinations that may greatly exceed environmental concentrations, may be combined in ratios that do not occur in natural streams subject to metal contamination, and may not have included concurrent testing of single‐metal and mixture exposures (Farley et al. 2015; Meyer et al. 2015).

Water quality criteria and risk assessments commonly rely on species sensitivity distributions (SSDs) compiled from laboratory toxicity data sets. Reliable values for aquatic insects in SSDs are scarce, comprising approximately 1 to 2 of the taxa out of the 20 to 30 chronic taxa values which commonly populate metal SSD data sets (Brix et al. 2005; Mebane 2006; DeForest and Van Genderen 2012; Croteau et al. 2019). For instance, Brix et al. (2011) found only 9 laboratory toxicity studies testing aquatic insects from 2000 to 2010 compared to hundreds of papers published with other aquatic taxa over the same time frame. Further, much of the published aquatic toxicity data with metals in aquatic insects may be of little environmental relevance if the tests were short term (acute); if larvae were exposed in unfed, water‐only exposures (Poteat and Buchwalter 2014); or if field‐collected, larger, late‐instar insects were tested (Kotalik and Clements 2019). In the wild, this imbalance is reversed. For example, in a study in forested mountain streams, insects outnumbered fish 10000 to 1, the species richness of insects in reference streams exceeded 40 compared to 0 to 5 species of fish, and the total biomass from all aquatic insects was comparable to that of all fish, ranging between 1 to 10 g/m^2^ (Mebane et al. 2015).

In the wild, aquatic organisms will be exposed to metals both through diet and through aqueous (gill) exposures, the respective importance of which has been the subject of much debate. Laboratory accumulation studies of dietary or waterborne metals have shown that, at least for some metals and settings, most whole‐body accumulation in insects is dominated by dietary sources (Munger and Hare 1997; Xie et al. 2010; Brix et al. 2011; Cain et al. 2011; Kim et al. 2012; Wanty et al. 2017). Thus, laboratory toxicity testing of aquatic insects following standard methods that only expose insects to waterborne metals effectively expose insects to much lower metal loads than would result from the same waterborne concentrations in field settings. However, although some studies have shown that total accumulation was dominated by dietary metals, whether toxicity primarily results from dietary or aqueous exposures is uncertain because few studies have linked metal accumulation to toxicity in aquatic insects (Brix et al. 2011). Further, aquatic insects may have slower responses (weeks) to metals compared with zooplankton or fish (hours to days; Hook and Fisher 2001; Mebane et al. 2012, 2017; Poteat and Buchwalter 2014). For instance, with mayflies fed Zn‐spiked diatoms, detoxification mechanisms were estimated to have only activated after 10‐d exposure, and total Zn body burdens peaked at approximately 25d (Wanty et al. 2017), suggesting that shorter‐term exposures might miss toxic responses to Zn.

For several years, we have worked on experimental and modeling approaches using field data that might inform some of these limitations. These data sets include chronic metal tissue accumulation and apparent responses of freshwater communities to chronic metal mixtures from field surveys of benthic invertebrates and zooplankton (Schmidt et al. 2010, 2011; Balistrieri and Mebane 2014; Balistrieri et al. 2015). However, attributing observed patterns in field data to specific metals or mixtures through statistical fitting is uncertain. We have built on these initial efforts with controlled exposures of aquatic insect communities in matched single‐metal and metal mixture treatments. In our first experiment with this project (experiment 1), we exposed naturally colonized aquatic insect communities to Zn, Cd, and Cd+Zn mixtures for approximately 30d. Aquatic insect taxa richness and abundance declined in concentration‐dependent patterns, particularly so for mayflies, at concentrations far lower than those causing mortality in traditional acute toxicity tests (Mebane et al. 2017). However, large differences between targeted and achieved dissolved metal concentrations suggested that metals could be accumulating in periphyton, which in turn could be an important exposure route. The possibility of substantial accumulation of metals by periphyton in the experimental streams raises the question of whether observed effects should be attributed to direct toxicity from water or dietary toxicity through periphyton.

In the present study, we replicate and expand on our Zn and Cd experiment with 2 more artificial stream experiments which tested mixtures of Cd, Cu, Ni, and Zn: 1) experiment 1 used Zn, Cd, Zn+Cd (July–September, 2013; reported in Mebane et al. 2017); 2) experiment 2 used Zn, Cd, Cu, Zn+Cu, Zn+Cu+Cd (August–October, 2014; present study); and 3) experiment 3 used Zn, Cu, Ni, Zn+Ni, Zn+Ni+Cu (July–September, 2015; present study).

Whereas in experiment 1 only dissolved metals were measured, in experiments 2 and 3, metal accumulation was measured in sediments, periphyton, and *Brachycentrus* sp. caddisflies. Periphyton (also referred to as “biofilms” or “aufwuchs” in the literature) is ubiquitous in natural streams, coating nearly every substrate. Periphyton is a food source for aquatic insects and is procedurally defined as material accumulating on submerged surfaces which includes living algae and bacteria, dead organisms, and previously suspended organic and inorganic materials such as Fe and Mn oxides. Trace element accumulation in periphyton is usually greater than that in other aquatic compartments, such as macrophytes, sediment, insect, or fish tissue (Newman and McIntosh 1989). Tissue residues from caddisflies may be useful indicators of the actual bioavailability of metals to aquatic insects because they readily accumulate metals in their tissues yet are highly tolerant of elevated metals (Schmidt et al. 2011; Rainbow et al. 2012). In field settings, caddisfly tissue residues have in turn been good predictors of other community‐level effects such as loss of sensitive species (Cain et al. 2004; Schmidt et al. 2011; Mebane et al. 2015).

**Figure 1:**
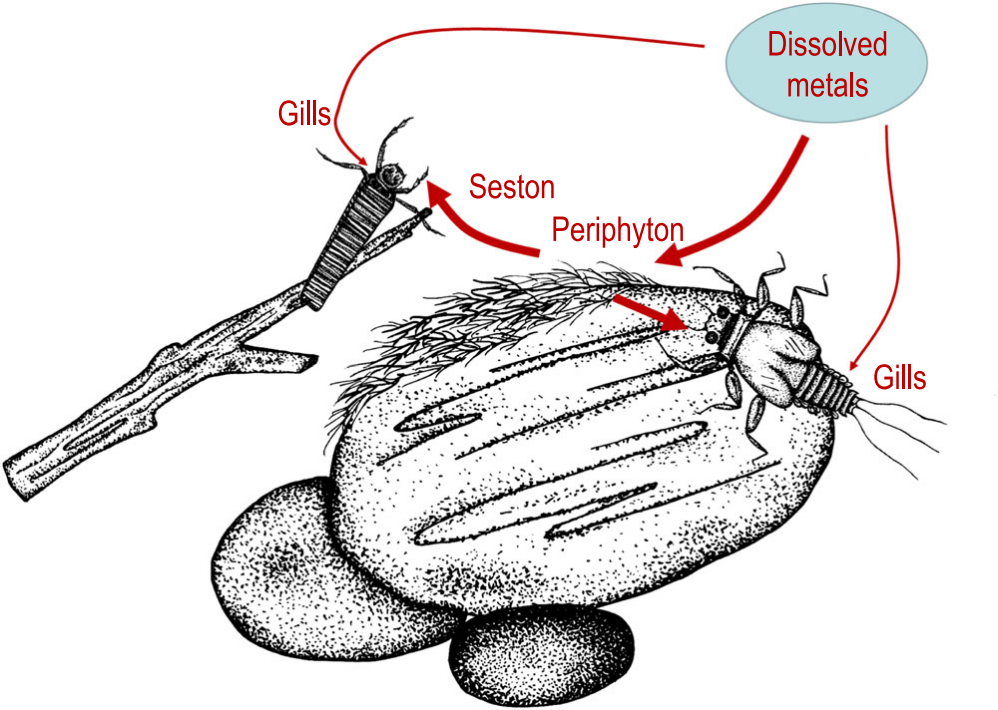
Concepts of periphyton‐mediated pathways of exposure of bioavailable metals to stream insects. Dissolved metals are strongly taken up by the living and dead organic material within periphyton. Mayflies and other grazing insects accumulate metals from feeding nearly exclusively on the metal‐enriched periphyton. Periphyton dislodged from the substrate enters the drift with particles captured and eaten by filter‐feeding or collector‐feeding taxa such as caddisflies. Dissolved metals are also taken up directly through the gills. (Commissioned artwork by Amy McMahon.)

Our conceptual model for concurrent metal exposures to aquatic insects through waterborne and dietary routes involves metals entering an experimental stream in dissolved form and sorbing to sediment substrates as well as active uptake by periphyton. Aquatic insects will be exposed to dissolved metals through their gills and dietary metals through grazing on periphyton (mayflies) or for caddisflies, collecting particles from the drift (Figure 1).

## METHODS

Our basic experimental design has been previously described in more detail (Mebane et al. 2017; Schmidt et al. 2018). Briefly, plastic trays filled with precleaned gravel were colonized by periphyton and macroinvertebrates in the Cache La Poudre River (Colorado, USA). After approximately 30 to 45d, the trays were transported to the laboratory and placed in artificial streams. The laboratory was an indoor artificial stream system with 36 experimental units (Aquatic eXperimental Laboratory [AXL]). Each unit was a 13‐L plastic bucket with a 13‐cm standpipe covered in 2‐mm mesh, a center‐perforated recirculation drain pipe covered in 2‐mm mesh, and a pump (capacity 1325 L/h) that recirculated water to simulate a riffle environment. When empty, the volume of the buckets below the drain pipe was approximately 6 L, but displacement from rock trays reduced the effective water volume to approximately 4 L. Peristaltic pumps continuously delivered spiked river water to the experimental streams at 2 mL/min and clean river water at 2 mL/min for approximately 2 daily volume replacements. The experimental streams were kept in 4 temperature‐adjusted water baths to maintain suitable temperatures of approximately 15 °C.

Four days before test initiation, Cache La Poudre River water from the colonization site was pumped streamside into a 2840‐L high‐density polyethylene tank and trucked to the AXL lab in 2 trips. The clean river water was stored in the laboratory in two 2270‐L polyethylene tanks, which was sufficient volume to complete the tests with a single collection.

For the 2014 Cu, Cd, and Zn test, the trays were colonized for 31d from 2 September to 3 October 2014 and exposed to metals for 30d from 5 October to 4 November 2014. For the 2015 Zn, Cu, and Ni test, the trays were colonized for 41d from 28 July to 7 September and exposed to metals for 30d from 8 September to 8 October 2015. To further evaluate control performance, in addition to the laboratory controls, the 2015 test included “river controls,” replicate trays that were left in place in the river and collected on the same day that the laboratory tests were ended.

### Metal treatments

**Figure 2:**
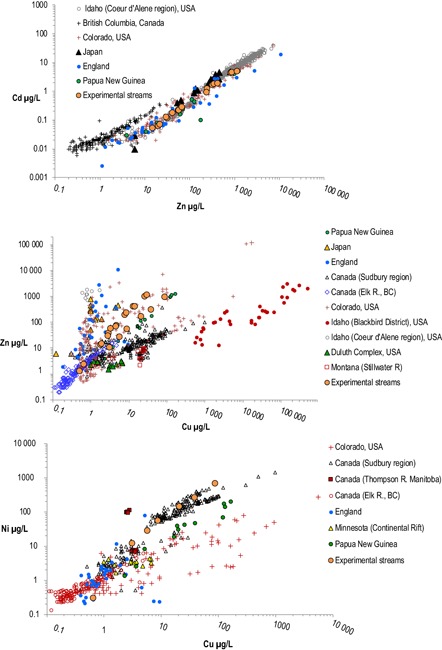
The toxicity of metals in mixtures is a function of both the concentrations and relative ratios of the mixtures. However, in natural waters, ratios of dissolved metals vary depending on the underlying mineral deposit types. Concentrations of Zn and Cd are nearly always strongly correlated, with ratios plotting in tight bands. Although with much more scatter than Cd and Zn, Cu and Ni appear to commonly be correlated across different deposit types. In contrast, Cu and Zn ratios vary tremendously with geographic regions and could be either Cu‐ or Zn‐dominated.

We selected target single and mixture ratios and concentrations to be within concentrations and ratios that actually occur in nature (Figure 2). Within these bounds, mixture ratios were also selected so that no single metal would overwhelm the mixture toxicity response. Zinc was used as a reference metal based on our earlier modeling work (Balistrieri and Mebane 2014; Balistrieri et al. 2015) and our testing in experiment 1, targeting a Zn:Cd ratio of 125:1 (Mebane et al. 2017). Target ratios for experiment 2 were Zn:Cu, 16:1; Zn:Cd, 200:1; and Zn:Ni, 2:1. For experiment 3, the target ratios were Zn:Cu, 8:1; Zn:Ni, 2:1; and Cu:Ni 1:4.

To maximize our test concentrations and mixture combinations, we adopted a “regression” design in which we replicated controls only, with one stream within each of the 4 water baths randomly assigned as controls. Treatment streams were not replicated so that by testing 6 treatments per series, the results would be more amenable to analysis by regression, which may provide more meaningful interpretation of results than just whether a treatment is “significantly different” from the controls (Liber et al. 1992; Cottingham et al. 2005). Replicating the controls allows calculating confidence intervals on mean control responses, which allows us to infer that treatment responses that fell within the 95th confidence interval of controls were similar to control responses (Di Stefano 2004; Belia et al. 2005).

Concentrated, 500‐mL stock solutions were prepared with a CdCl_2_‐hydrate 0.0005 M solution (Mallinckrodt, American Chemical Society grade), CuSO_4_‐pentahydrate, NiCl_2_‐hydrate, and ZnSO_4_·7H_2_O 0.05 M solution (Aldrich; 99.995% trace metal basis) and deionized water and acidified with ultrapure nitric acid. An appropriate volume of stock solution was added to each polyethylene carboy (head tank) containing 20 L of clean river water to give a final carboy concentration 4 times the desired nominal exposure concentration. Each carboy was the source of spiked Poudre River water for each stream. Four control streams received only clean river water (4 mL/min). The 4 mL/min dose rate results in close to 1.4 volume additions per day, continuously added and mixed vigorously with water already in the experimental stream. At that rate, each carboy had a capacity of 6.6d, but 2 sets of carboys were used sequentially, with a carboy refilled and spiked as needed. The initial filling of the streams and mixing of the stock solution into the carboys was 3d prior to the introduction of the colonized trays into the streams. We used an acclimation and concentration ramp‐up dosing strategy to avoid a shock response from transferring colonized trays directly into metal exposures. Organisms were transferred from the river to the experimental streams on day 0 and were allowed to acclimate in clean river water in the experimental streams for 24 h before the treatment drips were started on day 1.

The contact time between the stock solution and the river water may be important in toxicity testing with Cu because Cu complexes strongly to dissolved organic matter (DOM) and Cu–DOM complexes have low bioavailability and toxicity. However, equilibration between Cu and dissolved organic carbon (DOC) is not instantaneous, and test designs that only mix Cu with dilution waters for short periods may not be equilibrated and may expose organisms to higher proportions of more toxic, uncomplexed Cu forms than if the systems were in equilibrium (Ma et al. 1999). The contact time to equilibrium varies depending on organic carbon and water characteristics and is faster for natural DOM than for commercial humic acid for strong metal binding sites and waters with high calcium. In a well water with low DOC concentrations (~0.6 mg/L), equilibrium was approached in as little as 5 min (Meyer and Adams 2010) to <1 to 2 h with high DOC and low suspended material (Achterberg et al. 2002). Still, although differences were not large, Kim et al. (1999) reported that Cu with a 6‐h hydraulic residency time was more toxic than Cu with a 24‐h residency time. Through our approach of using premixed carboys which had a 6‐ to 7‐d capacity and replacing approximately 75% of the volume in the streams daily, the minimum contact time between the Cu stock solution and the river water would have been more than 1d and was usually considerably longer.

### Water, periphyton, tissue, and sediment sampling and analysis

Twice weekly, temperature (°C) and pH were measured for one stream from each treatment (*n* = 12). On these same days, water samples were taken for analytical chemistry. For anions and alkalinity, 125 mL of water were filtered (25‐mm Acrodisc Supor filters, 0.45 µm pore size) and refrigerated. For cations, 30 mL of water were similarly filtered and acidified to pH 2 with ultrapure nitric acid. In addition, on days 5, 19, and 30, raw, unfiltered cation samples were collected from the selected 12 streams and acidified. At the US Geological Survey's Central Mineral and Environmental Resources Science Center (Denver, CO), anions were analyzed by ion chromatography, and cations were analyzed by 44‐element inductively coupled plasma‐mass spectrometry (ICP‐MS; Wolf and Adams 2015).

Approximately 20 to 25 mL of water were filtered (combusted Whatman GF/F filters, 0.7 µm pore size) and collected in clean, precombusted glass vials for DOC measurements. Samples of DOC were frozen and analyzed by the University of Washington Oceanography Marine Chemistry Laboratory (Seattle, WA, USA) with a Shimadzu TOC analyzer using the total organic combustion catalytic oxidation method. Each stream was sampled 4 times on a staggered schedule that provided evenly spaced samples per treatment. At the end of experiment 3, benthic chlorophyll a was measured in situ, 4 readings per stream, using a “BenthoTorch” hand‐held fluorimeter (Aberle et al. 2006; Kahlert and McKie 2014). Algal responses were not quantified in experiments 1 and 2, and the benthic spectrophotometer was acquired after noticing less growth on rocks and clearer water in the high‐metal treatments.

Throughout the experiments, emergence nets were checked daily, and emergers were aspirated and frozen in clean Falcon tubes. On day 32, all experimental streams were sampled for larval macroinvertebrates. The 4 colonization trays in each stream were emptied into a clean 20‐L bucket. The rocks were gently scrubbed, and the organic matter and invertebrates in the bucket were decanted, sieved (500 µm), and preserved in 70% ethanol. A total of 36 experimental stream samples were collected at the end of each approximately 30‐d test. Preserved larvae and adults were enumerated, identified to the lowest possible taxonomic unit (typically genus for insects and family for noninsects), and sized to the nearest 1 mm.

*Brachycentrus* caddisflies, periphyton, and sediments were collected for residue analysis at the end of the exposures. *Brachycentrus* was targeted for analysis because they were the only taxa that was consistently present in streams including the high metal–dosed streams and because their large size provided sufficient tissue mass for analysis of even a single individual. Periphyton was scraped from the walls of the streams, and visible aggregations were collected by forceps. Sediments were collected from the streams following removal of the cobble trays. Periphyton and *Brachycentrus* whole‐body metal concentrations were determined as in Schmidt et al. (2011), except our samples were digested using an industrial microwave. Sediment samples were analyzed by ICP‐MS following a 4‐acid total digestion as described by Church et al. (2012). Sediments were sparse and consisted of suspended sediments that settled out among the cobbles in the rock trays during colonization.

Laboratory quality control analyses included river water matrix spikes and standard reference materials (https://bqs.usgs.gov/srs/) in addition to analyses of a duplicate and blank samples that we included on each sampling date. To quantify recovery of metals, National Institute of Standards and Technology standard 1577b, bovine liver and National Research Council of Canada certified reference material DORM‐4 (dogfish liver) were digested with sample batches over the course of the present study.

### Data analyses and data management

The data generated from the testing shared characteristics of both field bioassessment surveys and classic, flow‐through toxicity testing. The approaches used to evaluate effects drew from both practices. The community tests were similar to classic toxicity tests in that the exposures were known and followed a geometric series. However, unlike classic toxicity tests, the starting numbers of organisms were unknown and variable, owing to the vagaries of the colonization of benthic organisms during the river colonization of the test trays. Although abundant counts of a taxon at test end unambiguously indicate that conditions did not cause mortalities, low counts or absence of a taxon could reflect either metal mortalities from the dosed metals or low counts from colonization variability or other limiting factors (Schmidt et al. 2012a). Low taxa counts were considered to be attributable to factors other than metals if higher counts at higher concentrations were present. Taxa with abundances that were too low or too variable in control treatments were also excluded from regression analysis. This was defined by excluding taxa for which the Poisson lower 95th percentile confidence limit was negative or if any control replicate count was zero. Poisson distributions were used for this purpose because of their suitability for count data (Ritz and Vliet 2009).

Effect concentrations of dissolved metals were estimated using log‐transformed exposure concentrations with nonlinear regression from the US Environmental Protection Agency's Toxicity Response Analysis Program (Erickson 2015). This supported calculating classic effects percentage concentration values for 50% effects (EC50) or other percentages (ECp) for some individual taxa that were common enough to be found in all streams and which responded to the metal treatments. ECp values for aggregate metrics, such as total mayflies and species richness were similarly calculated. Piecewise linear regression was also used (reported in Supplemental Data) because a feature of this approach is the distinct breakpoint between the nonresponsive and responsive data regions which produce no‐effect (EC0) concentration estimates (Mebane 2015). In instances where all‐or‐nothing responses occurred, partial effects were inadequate to calculate statistical confidence intervals. In those instances, intervals bracketing the highest concentrations with no apparent response and the lowest concentration with complete or nearly complete mortalities were considered to be the confidence limits of the ECp values.

Responses in mixture exposures were predicted using response addition based on the fitted responses to metals in the single‐metal exposures and then taking the product of the single‐metal fits to predict joint responses at each dissolved metal concentration of interest (Meyer et al. 2015; Mebane et al. 2017).

The relative sensitivities of organisms in the tests were contrasted to that of other organisms by incorporating the effects concentrations into comparable SSDs. Previously developed chronic SSD compilations for Cd, Ni, and Zn were used (Mebane 2006; European Chemicals Agency 2008; DeForest and Van Genderen 2012), and an original chronic SSD for Cu was compiled (Supplemental Data). The SSD for Ni was updated with the 7 chronic invertebrate results reported by Besser et al. (2011). The Ni and Zn SSDs were supplemented with baetid mayfly 14‐d subchronic test data reported by Soucek et al. (2020). The Cd, Cu, and Zn EC20 values from the present study were adjusted to the same water types of the underlying SSDs. For Cd, a hardness–toxicity regression adjustment was used and biotic ligand models (BLMs) were used with Cu and Zn (US Environmental Protection Agency 2007; DeForest and Van Genderen 2012). Because the complexity of the Ni SSD, which used different BLMs for different taxa groups, the comparison was to a SSD normalized to a case study water with the most similar characteristics to our test waters (Lake Monate, Italy), and our calculated ECp values were further proportionally adjusted using the Bio‐Met “user‐friendly” BLM tool with test pH, DOC, and calcium values (wca 2015). Additional details on the SSDs are given in the Supplemental Data.

Detailed data on experiments 2 and 3 are available in the figshare data repository (see *Data Availability* section). The complete data sets for this and related studies are also archived in the http://ScienceBase.gov data repository (Schmidt et al. 2019).

## RESULTS AND DISCUSSION

### Metal mixture ratios tested in the experimental streams versus real‐world ratios

The ranges of tested metal mixtures in the experiments were well within those commonly expected in ambient waters. A goal with mimicking realistic exposure conditions in the artificial streams was to reasonably match the ratios and concentrations of metals in streams affected by metal mining (or areas with mineralogical conditions favorable for mining). Metals in stream water dissolve from host rock in watersheds. Mining disturbances in watersheds accelerate natural weathering processes, increasing concentrations, but largely retain the natural geologically determined concentrations (Wanty et al. 2009; Schmidt et al. 2012b). These patterns are shown in concentration plots from several geographically diverse data sets (Figure 2). Zinc and Cd are strongly correlated across the data sets, with a median mass ratio near 200:1. This narrow natural range of Cd and Zn mixture ratios is linked to sphalerite (ZnS), the mineral that is the chief ore of Zn. Cadmium can substitute for Zn in the sphalerite crystal lattice, so sphalerite regularly contains small percentages of Cd, often approximately 0.3%. No such consistent ratio occurs with Cu. In nature Cu has 2 oxidation states (^2+^ and ^1+^), whereas Zn has only ^2+^; as result, they rarely appear in the same mineral together, unless they are both minor components substituted in some other mineral. As such, Cu:Zn ratios in nature can be exceptionally broad, ranging from 1000:1 to 1:1000 (Figure 2). Nickel commonly occurs in association with Cu, but ratios can also vary widely and may not match those in rocks. For example, in waters from England and from the Duluth Complex of the Continental Rift area of North America average Cu to Ni ratios were approximately 1:1, whereas in streams in Colorado, USA, Cu exceeded Ni by approximately 8 times on the average (Figure 2). In rivers in Japan, Ni exceeded Cu by approximately 2 times on the average and up to 100 times (Takeshita et al. 2019). These principles also hold for urban or agricultural watersheds, although specific ratios vary (e.g., Hurley et al. 1996; Göbel et al. 2007).

To have relevance to natural waters, studies of effects need to be conducted within the bounds that are actually encountered in the landscape. Although Cd+Zn ratios are tightly constrained in most waters, other metal mixture combinations can vary widely in ratios and concentrations (Figure 2). An implication of these patterns is that some metal combinations that might make interesting toxicological cocktails such as binary mixtures of Cd+Cu or Cd+Ni are of much less interest in environmental settings because of the tight association between Zn and Cd.

### Nondegradation of control communities

The translocated, captive insect communities maintained their structure throughout the tests. In experiment 2, “day 0” quality control trays were preserved for analysis immediately on removal from the river. Total insect abundance in the day 0 trays averaged 230 (±66, 95th percentile confidence intervals) versus 197 (±70) for the day 30 laboratory controls. Comparisons are per “stream,” that is, 4 sets of 4 trays from the river, same as in the laboratory streams. Taxa richness averaged 28.3 (±2.0) in the day 0 river controls versus 30.3 (±4.6) in the day 30 laboratory controls. For discrete taxa, the abundances in the day 30 laboratory controls of 4 of the 66 taxa occurring in the experimental streams were outside the confidence intervals of the day 0 controls.

Experiment 3 had more differences between the day 30 laboratory controls and the day 0 river controls, but the undisturbed river control community also changed appreciably over the 30‐d period. Taxa richness in the undisturbed river controls declined from 40 (±9.8) to 25 (±3.9), and overall abundance declined from 638 (±109) to 438 (±81) animals per “stream” in the river from day 0 to day 30, respectively. The taxa richness was similar between the day 30 river controls and the day 30 laboratory controls, with 25 (±3.9) versus 24 (±1.5) taxa, respectively, whereas abundances were higher in the day 30 river controls than the day 30 laboratory controls, with 438 ± 81 versus 331 ± 109 animals per stream, respectively. Laboratory control abundances of 6 of the 77 taxa occurring in experiment 3 were outside the confidence intervals of the day 30 river controls.

The captive, closed laboratory control insect community will eventually degrade and become dissimilar to natural stream communities because, unlike the open, river controls, the larval insects that die or metamorphose in the laboratory streams will not be replaced (Schmidt et al. 2018). However, these river versus laboratory control comparisons indicate that the 30‐d test period was not too long to maintain and test a captive stream insect community.

### Time dependence of aqueous exposures

**Figure 3:**
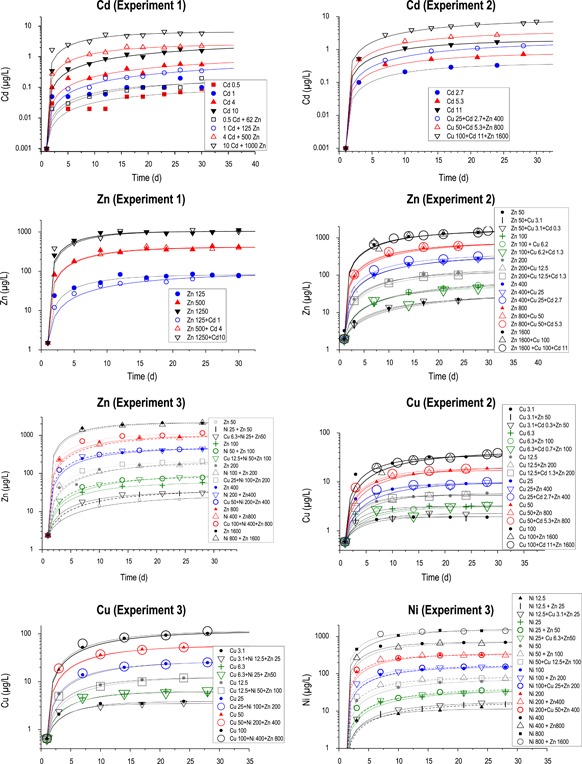
Time dependence of dissolved metal concentrations. Mixtures and single‐metal exposures matched nominal exposures, and except for the Cd experiments, mixtures and single‐metal exposures were very similar. Although the rates of increases tended to decline after approximately 5 to 7d, dissolved concentrations tended to continue to slowly rise throughout the exposures, particularly for Cd and zinc.

In all tests, dissolved metals increased sharply during approximately the first 5d and began to approach steady state by approximately day 10 in the Cu, Ni, and Zn exposures. In contrast, Cd increased steadily throughout the experiments. Complete steady state was uncommon, and concentrations were still slowly increasing at the end of most tests (Figure 3).

The exposures targeted identical concentrations of individual metals between the single‐metal and mixture exposures. Except for the Cd exposures, almost all of the fitted curves of measured concentrations versus time of single and mixture exposures plotted on top of each other. This indicates to us that, first, our dosing system combining high‐precision peristaltic pumps with frequent flow monitoring produced highly accurate and stable dosing. Second, for Cu, Ni, and Zn exposures, competition for binding sites on surfaces within the streams was not an important factor in the mixture exposures. With Cd, for equivalent dosing, the measured dissolved concentrations were consistently higher in the Cd+Zn and the Cd+Zn+Cu exposures (Figure 3A,B). This suggests that at common Cd:Zn environmental ratios (1:200; Figure 2), the Zn concentrations outcompete Cd for potential surface binding sites.

In experiment 2, DOC slowly declined from a mean of 3.75 (±0.37 SD) mg/L at the test start to 2.91 (±0.05) mg/L at the test end. The experiment 3 test conditions were similar, with Cu, Ni, and Zn concentrations approaching plateau concentrations near day 14 and DOC concentrations slowly declining from a mean of 3.5 mg/L at the start to 2.7 mg/L at test end (Supplemental Data). We presume the slow and steady declines resulted from microbial respiration in the holding tanks. All other measured characteristics were very stable during the tests (e.g., pH, temperature, major and trace ions; Supplemental Data).

Background concentrations of metals in river water were low for all measured metals but uncertain for Cd and Ni. In experiment 1 (Mebane et al. 2017), using diffusive gradients in thin films (DGTs), we measured background Cd at 0.001 µg/L; but DGTs were not used subsequently, and background Cd was reported as <0.02 µg/L in experiments 2 and 3. In experiment 1, background dissolved Ni was reported as 0.3 µg/L, whereas in experiments 2 and 3, reported values were <0.1 µg/L (Tables 1 and 2). All of these concentrations were well below those tested in external quality control certified or standard reference materials (Supplemental Data); thus, the true background river concentrations within these ranges are uncertain.

**Table 1:** Experiment 2 (2014), Zn, Cu, and Cd: Summary of aqueous exposures and larval abundances per stream^a^

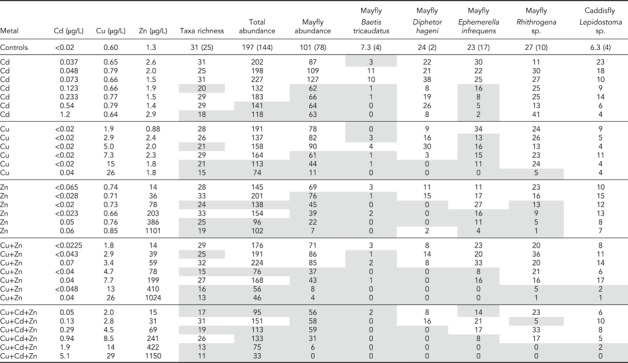

a For controls (*n* = 4), test‐wide averages are shown; numbers in parentheses are the lower of the lowest control replicate value or the lower 95th percentile Poisson confidence limit. For all other treatments, *n* = 1. Shaded boxes indicate that the value was less than the lowest control value and the lower 95th percentile confidence limit for the control mean. Average background dilution water conditions ± standard deviation: specific conductance 47 ± 1.6µS/cm, temperature 15.1 ± 0.4 °C, pH 7.5 ± 0.1, dissolved organic carbon 3.2 ± 0.28 mg/L. Major constituents in milligrams per liter: hardness as CaCO_3_, 18.1 ± 0.7; Ca, 5.3 ± 0.2; Mg, 1.2 ± 0.05; Na, 1.9 ± 0.2; K, 0.77 ± 0.02; SO_4_^–^, 2.9 ± 0.8; Cl^–^, 0.88 ± 0.2; alkalinity as CaCO_3_, 17.5 ± 1. Trace elements in micrograms per liter: Al, 8.6 ± 0.94; Cd, <0.02; Co, <0.4; Cu, 0.60 ± 0.096; Fe, <50; Mn, 0.43 ± 0.12; Ni, 0.10 ± 0.03; Pb, <0.2; Zn, 1.3 ± 0.5.

**Table 2:** Experiment 3 (2015), Zn, Cu, and Ni: Summary of aqueous exposures and larval responses^a^

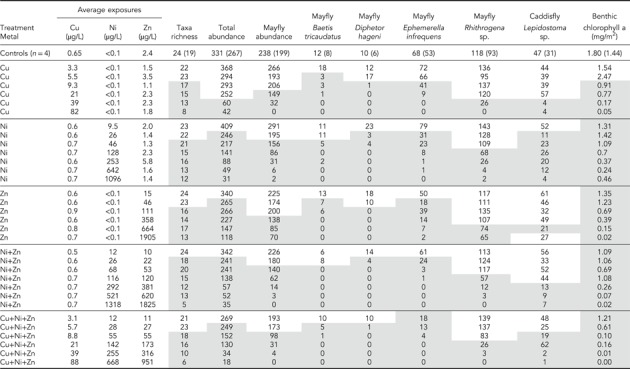

a Abbreviations and markings follow Table 1. Average background dilution water conditions, ± standard deviation: specific conductance 44 ± 2.3 µS/cm; temperature 15.2 ± 0.4 °C, pH 7.67 ± 0.13; dissolved organic carbon 3.0 ± 0.40 mg/L. Major constituents in milligrams per liter: hardness as CaCO_3_, 17.5 ± 0.7; Ca, 5.2 ± 0.2; Mg, 1.1 ± 0.04; Na, 1.4 ± 0.06; K, 0.61 ± 0.02; SO_4_^–^, 4.2 ± 01.3; Cl^–^, <0.6, alkalinity as CaCO_3_, 15.8 ± 0.6. Trace elements in micrograms per liter: Al, 7.4 ± 1.4; Cd, <0.02; Co, <0.02; Cu, 0.65 ± 0.14; Fe, <20; Mn, 0.47 ± 0.109; Ni, <0.1; Pb, <0.12; Zn, 2.4 ± 2.1.

### Metal accumulation in periphyton and caddisflies

**Figure 4:**
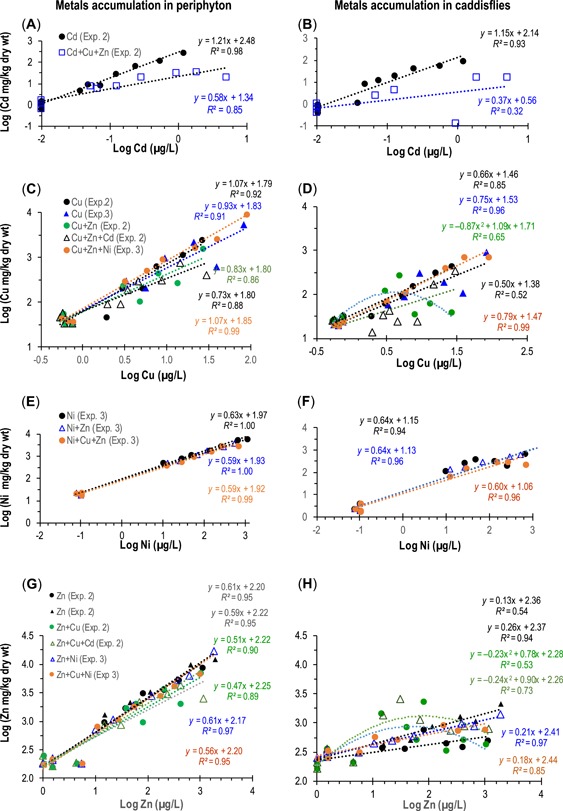
Metal accumulation in periphyton and caddisfly tissues from single metals or mixture exposures, as a function of dissolved single metals. Equations follow the order of the symbol legends. In the Zn and Cu series, periphyton accumulation for a given dissolved metal concentration tended to be lower in the mixtures than in the single‐metal exposures. The largest difference between single‐metal and mixture exposures was with Cd, in which Cd accumulation in the presence of Cu and Zn was an order of magnitude lower than Cd alone. In contrast, the presence of Cu or Zn had no discernable effect on Ni accumulation.

All metals were strongly accumulated into periphyton with Cd > Cu > Ni ≥ Zn, ranked according to their enrichment factors from dissolved concentrations (Supplemental Data). In the single‐metal exposures, metal accumulations in periphyton were very strongly correlated with dissolved metals, with log‐linear coefficients of variability (*R* ^2^) ranging from 0.91 to 0.99. In comparisons of accumulations in the single‐metal versus metal‐mixture exposures, periphyton accumulation for a given dissolved metal concentration tended to be lower in the Cu and Zn mixtures than in the single‐metal exposures. The most striking difference between single‐metal and mixture exposures was with Cd, in which Cd accumulation in the presence of Cu and Zn was an order of magnitude lower than Cd alone (Figure 4A). In contrast, the presence of Cu or Zn had no discernable effect on Ni accumulation (Figure 4E,F). Qualitatively, these patterns are consistent with the patterns in dissolved metal concentrations, with nearly identical concentrations in the single‐metal and mixture exposures, except for Cd.

For all metals, accumulated periphyton concentrations were much higher than those for the caddisfly *Brachycentrus* (Figure 4). Except for Ni, caddisfly tissues were higher than corresponding sediment accumulations from the same treatments (Supplemental Data). Metal accumulation was much more variable in caddisfly tissue than in periphyton (Figure 4). As with periphyton, the most pronounced difference in tissue accumulations between single‐metal and mixed‐metal exposures was with Cd. The slope of the dissolved Cd versus tissue Cd accumulation regression was much steeper for the single Cd exposure than the Cd+Cu+Zn exposure (Figure 4B). As with periphyton, Ni accumulations in caddisfly tissue closely tracked dissolved Ni, with *R* ^2^ values of 0.95 or greater; and there were no obvious differences in accumulations from the single‐ versus mixed‐metal exposures (Figure 4F).

In contrast, some Cu and Zn series showed highly variable accumulations in caddisflies. Two Zn series had reversals of expected accumulation patterns, with parabolic patterns (positive slopes with dissolved Zn at low concentrations, switching to negative slopes at higher concentrations; Figure 4H). Reversals in the expected patterns of increasing dissolved metals and increasing insect tissue concentrations may reflect reduced feeding as the caddisflies sicken in the high‐metal treatments or reflect a decline in dietary exposure with declining availability of living algal biomass as reflected by the declines in chlorophyll a in the high metal concentrations.

### Larval responses to single‐metal exposures

Across the experiments, quantitative effects concentrations could be estimated for 10 common taxa that showed concentration–response patterns. An additional 5 common taxa had no apparent effects at the highest exposure concentrations. Many other taxa occurred too infrequently to make specific inferences of sensitivity or tolerance. Selected responses are summarized in Tables 1 through 3, and more detailed information and counts of other taxa are given in the Supplemental Data.

**Table 3:** Summary of larval effect concentrations for individual taxa and aggregate metrics with credible concentration responses in the 30‐d tests: 50% effect concentrations

Taxa or metric	Group	Cd EC50 (µg/L)	Cu EC50 (µg/L)	Ni EC50 (µg/L)	Zn EC50 (µg/L)
*Baetis tricaudatus*	Ephemeroptera/Baetidae	0.38 (0.16–0.85)^a^	2.2 (0.6–7.3)^b^ ^,nc^	44 (38–52)^c^	75 (8–705)^a^
		0.11 (0.07–0.13)^b^ ^,nc^	4.9 (3.3–21)^c^ ^,nc^		13 (1.3–78)^b^
					79 (34–185)^c^
*Diphetor hageni*	Ephemeroptera/Baetidae	1.08 (0.48–2.4)^a^	6.7 (5.0–7.3)^b^ ^,nc^	22 (9.5–26)^c^ ^,nc^	39 (2–65)^a^ ^,nc^
			7.5 (5.3–9.3)^c^ ^,nc^		39 (1.3–78)^b^ ^,nc,vc^
					57 (15–111)^c^ ^,nc^
*Drunella doddsi*	Ephemeroptera/Ephemerellidae		63 (39–82)^c^ ^,nc^	30 (26–46)^c^ ^,nc^	309 (117–8220)^c^
*Drunella grandis*	Ephemeroptera/Ephemerellidae	>1.2^a^, ^b^	22 (20–24) ^c^	88 (59–132)^c^	>866^a^
					436 (217–876)^b^
*Ephemerella infrequens*	Ephemeroptera/Ephemerellidae	0.37 (0.11–1.2)^a^	6.9 (1.9–26)^b^	27 (17–43)^c^	184 (71–473)^a^
		0.19 (0.10–0.34)^b^	10.5 (9.2–12)^c^		394 (111–1392)^b^
					130 (36–467)^c^
*Epeorus longimanus*	Ephemeroptera/Heptageniidae	>1.1^a^			59 (21–169)^a^
*Rhithrogena* sp.	Ephemeroptera/Heptageniidae	1.2 (0.4–4.2)^a^	25 (15–26)^b^	129 (91–181)^c^	930 (568–1520)^a^
		>1.2^b^	31 (21–39)^c^ ^,nc^		65 (36–116)^b^
					1796 (564–5712)^c^
*Paraleptophlebia* sp.	Ephemeroptera/Leptophlebiidae	>1.1^a^	31 (21–39)^c^ ^,nc^	71 (23–218)^c^	316 (120–836)^a^
					115 (58–226)^c^
*Arctopsyche grandis*	Trichoptera/Hydropsychidae		>82^c^	>253^c^	>1100^b^
*Brachycentrus occidentalis*	Trichoptera/Brachycentridae	>1.2	>82^c^	>642^c^	>1100^b^
*Lepidostoma* sp.	Trichoptera/Lepidostomatidae	0.68 (0.13–3.4)^b^ ^,vc^	31 (21–39)^c^ ^,nc^	108 (20–614)^c^	>1008^a^
*Pteronarcella badia*	Plecoptera/Pteronarcyidae	>1.2^b^	>26^b^		
*Atherix pachypus*	Diptera/Athericidae	>1.2^b^	>82^b^	>1096^c^	>1905^c^
*Rheotanytarsus* sp.	Diptera/Chironomidae				337 (126–902)^a^
*Lebertia* sp.	Acari/Lebertiidae	>1.2^b^	>82^b^	>1096^c^	>1905^c^
Taxa richness (EC20 values)	Aggregate	>1.2^a^, ^b^	7.7 (0.4–150)^b^	53 (7–392)^c^	378 (167–856)^a^
			6.8 (1.2–39)^c^		653 (123–3475)^b^
					107 (46–358)^c^
Total mayflies	Aggregate	0.69 (0.28–0.67)^a^	13 (7.1–23)^b^	66 (37–117)^c^	160 (62–407)^a^
			24 (16–35)^c^		65 (7–656)^b^
					455 (156–1526)^c^
Periphyton chlorophyll a	Aggregate	Not tested	15 (7–33)^c^	73 (32–170)^c^	81 (38–170)^c^
USEPA chronic aquatic life criteria	Average conditions	0.20	8	12	26
EU EQS	(using Bio‐Met)	0.19	15	7	24

^a^ Experiment 1, 2013.

^b^ Experiment 2, 2014.

^c^ Experiment 3, 2015.

The US aquatic life criteria and European Union environmental quality standard calculations are explained in the Supplemental Data.

EC50 = 50% effect concentration; EQS = environmental quality standard; nc = not calculable, inadequate partial effects to calculate statistical confidence intervals; the intervals shown are the bracketing highest concentrations with no apparent response and the lowest concentration with complete or nearly complete mortalities. USEPA = US Environmental Protection Agency; EU = European Union; vc = effect concentrations should be considered tentative owing to high control variability.

With Cd in experiment 2, the most sensitive responses were from the mayflies *Baetis tricaudatus* and *Ephemerella infrequens* and the caddisfly *Lepidostoma* sp., with EC50s of approximately 0.2 to 0.5 µg/L (Tables 1 and 3). No other taxa showed obvious responses up to the highest concentration tested (~1 µg/L), although the lower overall taxa richness in the highest concentration suggested that some of the less common taxa were affected.

With Cu, in 2014 (experiment 2), the mayflies *Ephemerella*, *Diphetor*, and *Baetis tricaudatus* had the most sensitive responses, with EC50s of approximately 5 to 7 µg/L. Other than *Rhithrogena* sp., with an EC50 of approximately 25 µg/L, no other taxa had obvious responses up to the highest exposure (25 µg/L; Table 1). Similar responses were observed in the 2015 (experiment 3) Cu test for *Baetis*, *Diphetor*, and *Ephemerella*, with Cu EC50s at approximately 7 µg/L and with *Rhithrogena* at approximately 30 µg/L Cu (Table 2). In addition, in experiment 3 concentration responses were observed with additional taxa, including the caddisfly *Lepidostoma* and the mayflies *Drunella grandis*, *Drunella doddsi*, and *Paraleptophlebia* (Table 3).

With Ni in experiment 3, approximately the same rank order in taxa sensitivity was observed as with Cu, with the lowest EC50s obtained for *Ephemerella* and *Diphetor* of approximately 25 µg/L, with less sensitive responses from the mayflies in the genera *Baetis*, *Drunella*, *Rhithrogena*, and *Paraleptophlebia* and the caddisfly *Lepidistoma*.

Zinc was the only metal tested in all 3 of the 2013, 2014, and 2015 experiments. Response patterns were generally similar to those of the other metals with the exception of the mayfly *Rhithrogena*. Although as a group the mayflies are generally sensitive to metals, *Rhithrogena* was among the most resistant mayflies to Zn in 2013 and 2015. In those tests, major declines only occurred at the highest concentrations tested, with Zn EC50s of approximately 930 and 1800 µg/L, respectively (Mebane et al. 2017; Table 2). Yet, in 2014 a Zn EC50 of approximately 61 µg/L was obtained with consistent concentration‐dependent declines. No other taxa showed such pronounced differences in sensitivity to Zn or any other metal or combination of metals across the experiments.

Several common taxa showed no responses within the tested ranges of metal exposures. These taxa included the caddisflies *Arctopsyche grandis* and *Brachycentrus* sp., the stonefly *Pteronarcella badia*, the predatory dipteran *Atherix*, and the aquatic mite *Lebertia*. At least at the larval stage, these taxa were highly tolerant of the tested metals. The caddisfly *Brachycentrus* was consistently abundant and resistant to the metal exposures (present in 71 of 72 streams across the 2 tests; Supplemental Data). Because of their abundance, resistance, and large body size, this genus was targeted for tissue collection.

Other taxa may be sensitive to metals, but responses were ambiguous because of low or patchy numbers. For instance, in experiment 3, the chironomid midge *Polypedilum* was present in all controls and low‐metal treatments and was absent in all high treatments. However, in many cases only 1 or 2 individuals per stream were present, making any quantitative concentration effect estimates too uncertain to report.

Algal biomass in periphyton, measured as chlorophyll a showed consistent declines with increasing metal exposures in all test combinations and was one of the more consistently sensitive test endpoints measured (Table 2). Except for the 2 lowest Cu exposures, chlorophyll a densities were lower than mean control densities in all metal exposures; and in the more extreme mixture exposures, algal densities were suppressed to near detection levels. Effect concentrations for chlorophyll a declines were similar to those for the most sensitive mayflies (Table 3).

### Larval responses to metal mixtures

**Figure 5:**
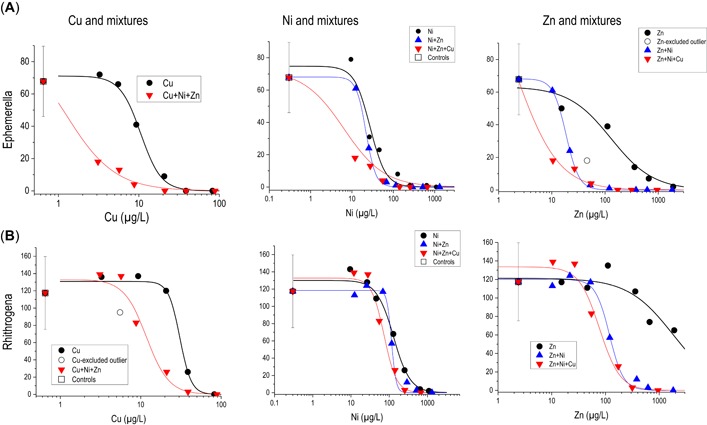
Responses of *Ephemerella* (**A**) and *Rhithrogena* (**B**) mayflies to Cu, Ni, and Zn in single‐metal or mixture exposures, plotted as a function of single metals. Data from experiment 3.

On an absolute toxicity basis, a given metal concentration was usually more toxic in the presence of other metals than if it were exposed individually (Figure 5). The exception was *Rhithrogena* responses to Ni and mixtures where Ni appeared to dominate the toxic response and the presence of Cu and Zn had little additional effect. Because the exposures were successful at replicating the concentrations between single‐metal and mixture exposures (Figure 2), the responses can be directly compared between the concurrent single and mixture exposures. In both experiments, the mixture exposures produced “something from nothing” (e.g., Versieren et al. 2016). That is, low metal concentrations that individually produced no apparent adverse effects did produce substantially lower mayfly abundances when exposed to similar concentrations jointly in the concurrent, lowest triple‐mixture exposures (Tables 1 and 2).

On a relative toxicity basis, the joint toxicity of the 5 tested metal mixture combinations tended to be less than additive when evaluated on a response‐addition basis for total mayflies. We focus our evaluations of mixture responses relative to single‐metal responses on the total mayflies metric, which reflects a generally metal‐sensitive, major component of the insect community (Tables 1 and 2).

**Figure 6:**
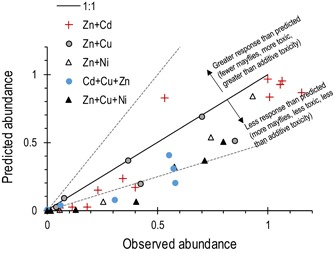
The joint toxicity of metal mixtures tended to be approximately additive on a response‐addition basis for Cu and Zn mixtures and less than additive for all other combinations. The solid line is the 1:1 line representing perfect agreement between predicted and observed abundance. Dashed lines indicate a 2 times difference from the 1:1 line. Where observed mayfly abundance in the mixture exposures is greater than that predicted from response addition (plotted below the 1:1 line), we interpret the mixture toxicity to be less than additive and vice versa.

Mixture toxicity by response addition was predicted by first fitting total mayfly response curves to the single‐metal responses. Then for a given mixture combination, the additive responses are predicted as the product of the single‐metal response curves. The predicted additive responses of each of the 37 tested mixtures are compared to the observed actual responses as a scatter plot, where the solid line represents the line of perfect agreement (Figure 6). The toxicity of Cu+Zn mixtures was approximately additive in most (4 of 6) treatments, whereas for most other combinations, the observed reductions in mayfly abundance were less than the predicted responses, suggesting that the Cd and Zn mixtures generally produced less‐than‐additive joint toxicity. The absolute differences from additive toxicity were not large, with most of the observed responses falling within a factor of 2 of the response‐addition predicted responses.

The conventional approach to criteria compliance monitoring in most jurisdictions is to evaluate concentrations of those chemicals for which regulatory criteria have been established one by one. Approaches for evaluating the additive toxicity of mixtures through adding cumulative toxic units or related calculations (Clements et al. 2013; Meyer et al. 2015) have not to our knowledge been extended from the environmental science literature into regulatory policy. Even under the less‐than‐additive, antagonistic conditions that prevailed in our experiments, a triple mixture of different metals almost always had a larger effect than the effect of any single metal in that mixture. The logical implication of neglecting mixture toxicity is that criteria evaluated in isolation will provide less protection than intended by the common criteria development approaches that define safe or hazardous concentrations on the basis of protecting all but the most sensitive 5% of an SSD of chronic test data.

### Sensitivity of insects and algae to metals

**Figure 7:**
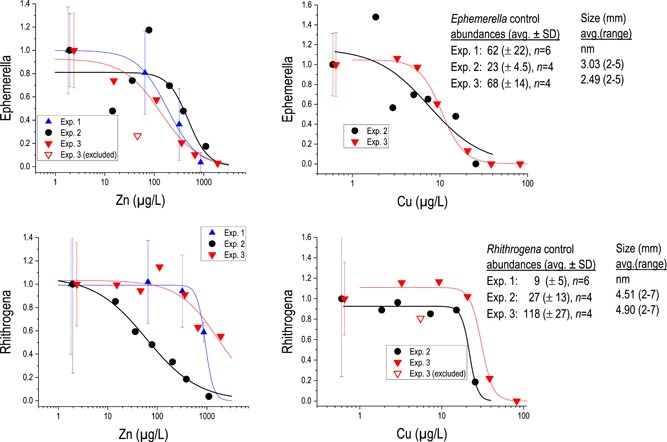
Intertest differences in responses of 2 common mayflies, *Ephemerella infrequens* and *Rhithrogena* sp., to Cu and Zn. Responses were normalized to control averages in each test; curves are from a 3‐parameter logistic model. Error bars show standard deviations of replicated exposures. Open symbols indicate low abundances that were judged not to have been caused by metals (based on occurrences of higher abundances at higher concentrations) and were excluded from curve fitting.

Consistent with experiment 1 (Cd+Zn), we recorded clear responses with several mayfly taxa and aggregate community metrics to the metal treatments. Year‐to‐year differences in response sensitivities between the tests were greatest for Zn, with *Diphetor* and *Ephemerella* EC50s varying by factors of 3 to 4, and *Rhithrogena* varied by a factor of 20. In contrast, Cu responses were highly repeatable across the 2 tests, within approximately a factor of 1.5 (Table 3). Factors driving the variability are not obvious. There were size differences between the tests, and more sensitive results were obtained in years with smaller average‐sized organisms. Smaller insects are generally more sensitive to metals than are larger/older ones (Kiffney and Clements 1996; Kotalik and Clements 2019; Cadmus et al. 2020). However, average sizes were not remarkably different between experiments 2 and 3, and the ranges were the same. *Rhithrogena* were also 10 times more abundant in experiment 3 than experiment 1 (Figure 7). Further, differences in sensitivity were not consistent with different metals. *Rhithrogena* was much more sensitive to Zn in experiment 2 but not so to Cu or Cd. Why individuals drawn from the same population of a species at the same locations, tested in similar chemical settings, become much more sensitive to one metal but not others is not obvious from the data collected. We suspect that subtle developmental differences may be at play, related to differing streamflow and river thermal conditions prior to testing, although more thorough exploration is warranted.

Results from our experimental streams tended to be considerably more sensitive than traditional water‐only tests but were not necessarily more sensitive than other mixed water and dietary exposures. With Cu, Clements et al. (2013) observed considerably more sensitive results than did we, in similar dilution waters, even though they used a shorter exposure duration (10 vs 30d). For example, their exposures produced an EC50 for *Rhithrogena* of 6 µg/L compared to 25 to 34 µg/L from our 2 tests. Responses of mayflies in aggregate as a group were more similar, with 50% reductions (EC50s) at approximately 10 µg/L in their test and 13 and 24 µg/L in our 2 tests (Table 3). Yet the sensitive *Rhithrogena* responses observed by Clements et al. (2013) differed greatly from earlier tests conducted in the same laboratory with similar methods. For instance, Buchwalter et al. (2007) only found reductions in mayfly taxa at considerably higher metal exposures than did Clements et al. (2013) in the same facility using similar methods. The test‐to‐test differences in both this earlier work and the present study are unexplained, but the influence of year‐to‐year environmental differences on the developmental timing is suspected.

The influence of exposure duration on the sensitivity of results appears to vary by metal and organism. As noted, 10‐d exposures may be sufficient to produce sensitive results with Cu, which was also observed in the experimental stream studies by Leland et al. (1989), which were reanalyzed by Brix et al. (2011). Soucek et al. (2020) obtained similar lowest‐effect levels for Ni and Zn when comparing full life cycle versus 14‐d subchronic tests, both initiated with <24‐h‐old larvae. In contrast, Clements (2004) found that 10‐d exposures to Zn or Cd+Zn mixtures produced few effects on the structure of insect communities, whereas we found marked reductions in overall taxa richness and the abundances of all mayfly taxa in similar concentration ranges but following approximately 30‐d exposures (Tables 1 and 2). Declines in taxa richness and mayfly abundances observed following 30‐d exposures to Cd+Zn mixtures in our experimental streams were similar to patterns observed across a gradient of Cd+Zn in mining‐affected natural streams (Mebane et al. 2017). In contrast, a comparison between responses in 10‐d experimental stream exposures and field conditions showed that the shorter experimental exposures underestimated effects of metals in field settings (Iwasaki et al. 2018). Although Iwasaki et al. (2018) cautioned that short‐term mesocosm tests may be better suited for testing ecological theories and informing the design of field studies than directly predicting adverse or safe conditions in the field, the 30‐d exposures (present study) may be more directly applicable to field conditions. In methods development testing, the community structure remained mostly intact for up to 35d of captivity (Schmidt et al. 2018); and in the present experiments, communities were similar between the captive laboratory controls and concurrent “river control” insect trays that were left in place in the river for the duration of the experiments (Supplemental Data).

Hickey and Golding's (2002) 34‐d Cu+Zn experimental stream study has similarities to the present study in terms of duration, metal ratios, and water chemistry. They reported their mixture results in terms of cumulative criterion units (CCUs), with an EC50 for reductions in mayfly abundance of 4.8 CCUs (Hickey and Golding 2002). Using the same versions of criteria described by Hickey and Golding, the Cu+Zn series from experiment 2 yielded an EC50 of 5.1 CCUs (Supplemental Data).

**Figure 8:**
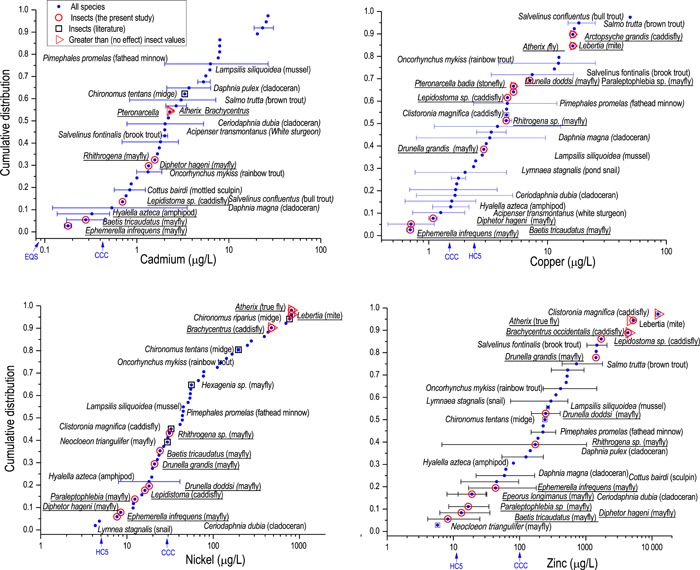
Overlays of the insect responses from the present study with chronic ranked species sensitivity distributions (SSDs) for all taxa. The range of responses from the experimental stream community exposures nearly spans the complete range of least to most tolerant taxa in the SSDs. Plotted values are mean species chronic 20% effect concentrations (except Ni with 10% effect concentrations), using the bioavailability adjustments to a common water type consistent with the underlying SSDs (see text). Error bars show ranges for taxa with more than one value available. Underlined taxa names are from the present study. CCC = US Environmental Protection Agency current chronic criterion concentration (details given in Supplemental Data); HC5 = hazardous concentration for 5% of species in a European Union Water Framework Directive SSD.

Mayflies were consistently sensitive across our tests, although they varied between taxa and sometimes year to year for the same taxa. The high sensitivity of *Ephemerella* to all 4 metals was expected based on physiological traits (Buchwalter et al. 2008) and is congruent with field observations (Clements 1994; Mebane et al. 2015). However, the highly sensitive responses of baetid mayflies across experiments to all 4 metals were incongruent with field patterns. In field studies of metal‐contaminated streams, *Baetis* sp. mayflies have been observed to be among the more tolerant of the generally metal‐sensitive mayflies (Clements 1994; Mebane et al. 2015). Similar to the present study, the baetid mayfly *Neocloeon triangulifer* was the most chronically sensitive taxa to Zn in a sensitivity distribution of 35 taxa, although it was near to the middle of the sensitivity distribution for Ni (Soucek et al. 2020; Figure 8). Life history characteristics likely contribute to *Baetis* persistence in streams (short life cycle, high propensity to drift), which could explain their quick recovery after pulse disturbances (Iwasaki et al. 2018). In contrast, the sustained presence of *Baetis* under constant metal pressure would not be easily explained by drift and short life cycles. At least some *Baetis* taxa can induce protective metal binding protein mechanisms, making them highly tolerant of metals (e.g., *Baetis thermicus*; Aoki et al. 1989). It is plausible that the *Baetis* taxa were sensitive to a novel metal stress, but surviving individuals acquired a tolerance to further exposures, which could explain why organisms were highly sensitive in novel laboratory exposures but could acclimate to and survive long‐term exposures in the wild (Clements 1999; Kashian et al. 2007).

Reductions in algal biomass as chlorophyll a were the most sensitive endpoint measured for Ni and Zn in experiment 3. The concurrent declines in algae and grazing mayflies suggests the plausibility of indirect effects on mayflies from reduced food availability. Indeed, if mayfly abundance is plotted as a function of algal biomass, it produces curves that look similar to concentration‐response curves (Supplemental Data, Figure S4). However, subtle differences in the patterns suggest that the declines are primarily attributable to metal toxicity rather than food limitation. Although some treatments show chlorophyll and mayflies declining to zero together, mayflies declined to zero in the Ni exposure whereas chlorophyll was still abundant; and in the Zn exposures, chlorophyll declined to near zero while mayflies were still abundant. *Brachycentrus* caddisflies, which primarily feed on algae and other organic particles in the drift, were largely unaffected by the treatments, suggesting that the food loss from reduced primary productivity had not yet become dire during the 30‐d exposures. In experiment 2, some chironomids were relatively common. The most abundant occurrence of *Rheocricotopus* (a small‐bodied, Orthocladiinae chironomid) was in the highest Zn treatment (1100 µg/L). This suggests that alternate food sources were sufficient. In natural streams, mayflies, Chironomidae, and *Hydropsyche* and Limnephilid caddisflies were all found to opportunistically feed on both dead plant detritus or living algae depending on availability (Gilpin and Brusven 1970; Koslucher and Minshall 1973). We would not expect plant detritus to have declined in the experimental streams with increasing metal treatments. The drastically reduced algal biomass does indicate that reductions in community tests and field settings are interactive and complex, and the mechanisms of toxicity (indirect or direct via aqueous and/or dietary exposures) are uncertain.

Whether metals would similarly cause sustained depression of chlorophyll a in the field would likely be situational. Lower periphyton chlorophyll a densities have been noted in zinc‐influenced streams (Courtney and Clements 2002; Carlisle and Clements 2003). In Cu‐dosed outdoor experimental streams, autotrophic production declined sharply; but heterotrophic production was less affected, and effects on overall periphyton biomass were minimal (Leland and Carter 1985). Nevertheless, the mayflies *Baetis* sp., *Ephemerella infrequens*, *Drunella* sp., and *Paraleptophlebia* sp. also showed declines in that study, consistent with the present results (Leland and Carter 1985; Leland et al. 1989). However, in other settings, metal exposures have resulted in both declines and increases in chlorophyll a. In the first 2 mo of an 18‐mo outdoor experimental stream exposure with Cu, chlorophyll a declined by 90% from controls in the highest Cu treatment (57 µg/L). This decline was followed by a rebound in the second year as grazing invertebrates declined and a Cu‐tolerant diatom took over the periphyton community (Roussel et al. 2007; Joachim et al. 2017).

Contaminants in aquatic ecosystems have been shown to cause indirect ecological effects such as changes in behavior, competition, and predation/grazing rate that can alter species abundances or community composition and enhance, mask, or spuriously indicate direct contaminant effects. These indirect effects can be as significant as or more significant than the direct toxic effects of a contaminant (Fleeger et al. 2003). When potential direct and indirect effects are strongly correlated, teasing them apart is difficult (Gardham et al. 2014; Rogers et al. 2016; Van Regenmortel et al. 2018). The uncertainty around the mechanisms of direct and indirect effects of metals on aquatic insect communities presents an obvious line of inquiry for further study.

At the larval stages, no non‐mayfly taxa were consistently sensitive to metals. *Rheotanytarsus* and *Micropsectra/Tanytarsus* sp. (Diptera) declined in some tests with increasing metals but were inconsistent or rare during other tests. *Lepidostoma* was the only caddisfly to respond to metals, declining with increasing Cu, Ni, and Cd but not Zn over the ranges exposed. This finding is similar to that of Nebeker et al. (1984), who found the caddisfly limnephilid responsive to Cu and Ni but not Zn in life cycle testing. *Lepidostoma* caddisflies were more sensitive to Cu and Ni in our 30‐d larval exposures than were the *Clistoronia* caddisflies in the full life cycle tests of Nebeker et al. (1984). This suggests that for some aquatic insect taxa, larval exposures may be a reasonable proxy for full–life cycle responses, analogous to assumptions that early–life stage tests with fish approximate full–life cycle responses. Similarly, short‐term exposures of marine invertebrates were presumed sensitive enough to protect for long‐term exposures; and thus, 4‐d or shorter tests are sufficient to represent chronic endpoints for some species that would be difficult to conduct full–life cycle tests with (Stephan et al. 1985). Such assumptions may not always be correct. For example, metamorphosis is a stressful life‐stage transition, and metal‐exposed insects may show reduced emergence even when effects on larvae were not detected (Nebeker et al. 1984; Schmidt et al. 2013; Wesner et al. 2014; Rogers et al. 2016).

Incorporating the results from the present study into SSD compilations shows the tremendous variation in metal tolerance of aquatic insects (Figure 8). Responses from these tests nearly span the entire distribution of existing data for all 4 metals, with the mayfly responses extending the sensitive tail of the distributions for Cd, Cu, and Zn. The new community responses incorporated directly into the SSDs could address the imbalance that could develop when testing of “new” species tends to target potentially sensitive taxa, which over time could skew the (debatable) concept that compiled laboratory SSDs are representative of the distribution of sensitivities in natural environments (Croteau et al. 2019). Further, these results refute analyses of toxicological databases which reported that aquatic insects were the most metal‐tolerant group of the aquatic macroinvertebrates (Malaj et al. 2012).

We recognize that there may be considerable debate on how or even whether effect concentrations should be calculated from community tests such as those described in the present study. Although the concept of deriving EC50 values, other effect percentile concentrations, or no‐effect concentrations from regressions of multiple taxa within a community test is not novel (Liber et al. 1992), the approach is used less than analysis of variance‐type testing. Analysts accustomed to working with data from highly controlled laboratory toxicity tests with cultured, “standard” species may be put off by the variability inherent to naturally colonized mesocosm tests. Acknowledging concerns that our 30‐d larval exposures may not be long enough to capture truly “chronic” or critical aquatic insect life‐cycle stages (Schmidt et al. 2013; Kotalik and Clements 2019), we think that the comparisons with chronic tests with other species (Figure 8) and with field surveys (Mebane et al. 2017) support the realism of the 30‐d mesocosm exposures.

Sensitivity rankings across repeated tests within the present study were generally similar, which increases our confidence in the results. For example, viewed in isolation, some tests with the mayflies *Baetis* and *Diphetor* could be discounted because numbers in controls were variable and low (often fewer than 10 per stream), with few partial effects in the metal treatments. Yet over the course of our 4 yr of testing, we conducted 18 separate exposure series with different combinations of 5 metals, and these 2 taxa had sensitive responses in 17 of 18 exposures (Schmidt et al. 2019). Together, the apparent realism in the data and general repeatability support arguments that standardized rules for compiling standardized test results need to be broadened when considering whether mesocosm and field data are “acceptable data” for criteria derivation or risk‐assessment purposes (Buchwalter et al. 2017).

### Waterborne and dietary metal exposures

**Figure 9:**
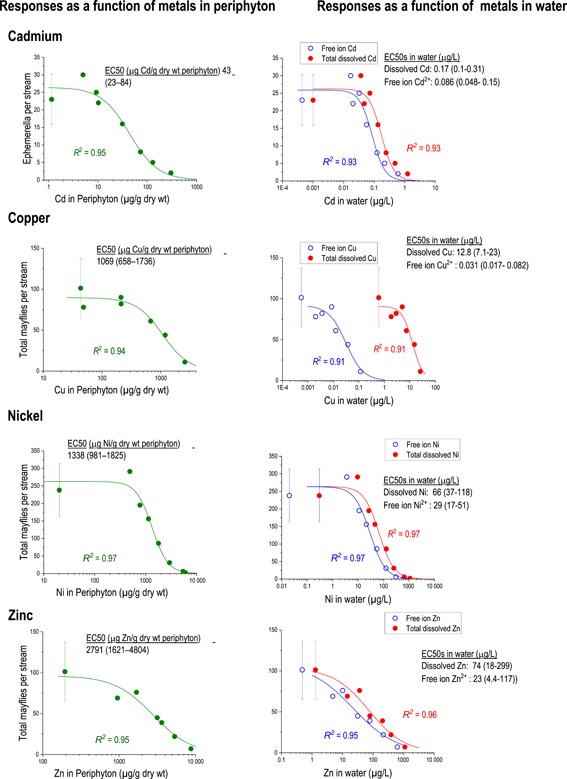
Examples of response curves of mayflies declines with Cd, Cu, Ni, or Zn concentrations expressed as functions of metals in periphyton, time‐averaged total dissolved concentrations, or the modeled free ion concentrations in the water column (data from experiment 2, except Ni from experiment 3). Error bars are standard deviations. EC50 = median effect concentration.

The strong correlations between dissolved metals and metals in periphyton raise the question of whether the biological responses should be attributed to dissolved metals, ingested metals, or both. To illustrate that conundrum, biological responses (declines in mayfly abundance) are shown as a function of either metals in periphyton or, more conventionally, dissolved metals (Figure 9). The patterns between the “dose–response” curves showing mayfly abundance declining with increasing metals in periphyton are very similar to the “concentration–response” curves, with mayfly declines as a response to dissolved metals. The strong correlations preclude making inferences directly from the data on the respective roles of dietary or dissolved metals in biological responses. Although metal toxicity and regulatory criteria are customarily interpreted based on dissolved metals in water, diet may account for the bulk of actual metal exposure to primary consumers in at least some settings (e.g., Croteau and Luoma 2007; Cain et al. 2011; Golding et al. 2013).

The response patterns show that dietary exposures (as periphyton concentrations) could explain metal toxicity at least as well as the dissolved metals (Figure 9). For the dissolved metals, the free ionic fraction of the dissolved metals is presumed to represent the more bioavailable fraction for physiologically active binding sites at the site of action (e.g., insect gill, plant cell wall, or other “biotic ligands”). With Cd, Ni, and Zn, the free ionic fraction dominated the solutions, representing over half of the total dissolved metals (modeled using the Windermere Humic Aqueous Model, assuming that unmeasured DOM equals 2 times the measured DOC concentrations [Lofts 2012]). With Cu, the free ionic fraction accounts for <1% of the total dissolved metal, with Cu–DOM complexes accounting for most of the dissolved fraction. This contrast illustrates the overwhelming importance of DOM for speciation calculations with Cu and the importance of modeling assumptions.

We are far from the first to observe strong correlations between dissolved metals, metals in periphyton, and metal residues in insects (Kiffney and Clements 1993; Hickey and Clements 1998; Harris 1999; Farag et al. 2007; Mebane et al. 2015). With Zn, Genter et al. (1987) suggested that monitoring periphyton metals could be more informative than monitoring metals in water, noting that Zn bound to periphyton was more reliable than total Zn in water for indicating Zn stress. The strong correlations between metals in periphyton and water imply that one could mask the effects of the other or that the dietary role of metal toxicity to insects may be unrecognized. In their “water‐only” exposure of *Hyalella* to Cd, Golding et al. (2013) showed that by simply feeding the organisms, dissolved metals quickly sorbed to the food. Thus, even in “water‐only” toxicity tests in which organisms are fed, much of the exposure could actually be dietary.

We limited the exposures to approximately 30d because we felt that longer exposures would exacerbate captivity effects and confound extrapolation of results to natural settings (Schmidt et al. 2018). However, this raises the question of whether relatively short‐term (30d) exposures are sufficient to approximate steady‐state conditions. The time‐dependent water concentrations (Figure 2) and other studies in which periphyton and insect concentrations were measured over time suggest that 30‐d exposures approach steady state. Using an experimental stream design similar to ours, Harris (1999) observed that periphyton reached quasi‐stable concentrations after approximately 2‐d exposure to Zn in experimental streams, and Zn concentrations in caddisflies appeared to plateau by approximately 10‐d exposures to water and periphyton in the experimental streams. Very similar results were observed from transporting caged caddisflies into a Cu‐ and Zn‐contaminated river (Tochimoto et al. 2003). In some cases, quasi‐steady‐state tissue accumulations can be much more rapid, such as 3d for *Hyalella* fed Cd‐enriched periphyton (Stephenson and Turner 1993).

Although many studies have shown that dietary exposures of metals may be more important than dissolved sources for overall body burdens in insects, we found few that linked dietary exposures to effects. Irving et al. (2003) exposed *Baetis tricaudatus* to Cd‐enriched periphyton mats (10 µg/g) and observed reduced growth after 13d. This was similar to the concentrations in periphyton at the curve break between no and severe effects to *Ephemerella* in experiment 2 (Figure 9). Xie et al. (2010) exposed the baetid mayfly *Neocloeon triangular* to Cd‐enriched periphyton in an approximately 30‐d test during summer and observed severe effects at periphyton concentrations of approximately 3 µg/g dry weight and above, which was 10 times lower than the lowest severe effects periphyton concentration in our tests (Supplemental Data). However, when they repeated the experiments in fall and winter at up to 8 µg/g dry weight Cd in periphyton, no effects were observed. Elevated Zn in the periphyton diet has been linked to reduced growth in the mayfly *Epeorus latifolium* (Hatakeyama 1989) and *Baetis tricaudatus* (Carlisle and Clements 2003). Wesner et al. (2017) exposed late instar *Baetis tricaudatus* collected from an urban stream to Zn in a similar experimental setup as used in the present study. No obvious effects on survival of late instars (i.e., adult emergence) were observed at periphyton Zn concentrations up to 12000 µg/g dry weight, whereas in our experiments no *Baetis* survived treatments with >5000 µg/g Zn in periphyton (Supplemental Data). These examples show that interpreting the effects of metals on insects in dietary exposures is complex and that factors such as season, developmental stage, exposure history, composition of the periphyton (Figure 4), and ratios of aqueous and dietary exposures may be important.

Returning to the 3 logical disconnects motivating the study, first, our data emphasize that mixture toxicity should not be ignored in risk‐assessment or criteria considerations, for even under less‐than‐additive (antagonistic) conditions, low concentrations can be toxic in combination even when none of the individual component concentrations was toxic. Second, the results show that even a single insect community test can produce effect concentrations that span the existing SSDs for the tested metals. For common insect taxa, effect concentration percentiles such as EC50 or EC20 values can be calculated in the usual fashion for toxicity testing. Third, metals were strongly accumulated by periphyton, and toxicity could be predicted well as a function of either exposure to metals in periphyton (a proxy for diet) or dissolved metals. This supports arguments that traditional, water‐only toxicity tests with aquatic insects indeed are environmentally irrelevant (Poteat and Buchwalter 2014).

Our data suggest that biological responses could be a function of exposures to metals through both waterborne and dietary exposures. In natural streams and in our experimental streams with concurrent water and dietary exposure routes, the relative influences cannot be directly teased apart. However, it has occurred to us that biodynamic modeling approaches could give insight to this question (Buchwalter et al. 2007; Cain et al. 2011). We intend to explore this approach in further work.

## Supplemental Data

The Supplemental Data are available on the Wiley Online Library at https://doi.org/10.1002/etc.4663.

## Disclaimer

The use of trade, firm, or product names is for descriptive purposes only and does not imply endorsement or disparagement by the US Government.

This article has earned an Open Data/Materials badge for making publicly available the digitally shareable data necessary to reproduce the reported results. The data are available at https://doi.org/10.6084/m9.figshare.8986676 and https://doi.org/10.5066/p9xxbsak. Learn more about the Open Practices badges from the Center for Open Science at https://osf.io/tvyxz/wiki.

## Supporting information

This article includes online‐only Supplemental Data.

Supporting information

## Data Availability

Supporting information is available via the figshare data repository (https://doi.org/10.6084/m9.figshare.8986676). The full data sets from these and related experiments are also available via the US Geological Survey ScienceBase repository at https://doi.org/10.5066/p9xxbsak (Schmidt et al. 2019).
