# Supplementary material for: Bioaccumulation and Toxicity of Cadmium, Copper, Nickel, and Zinc and Their Mixtures to Aquatic Insect Communities
Source: Environ Toxicol Chem. 2020 Mar 26;39(4):812–33. doi: 10.1002/etc.4663 (PMC7154727; doi:10.1002/etc.4663)
Supplement: Supplementary file 1 — Supporting information [file ETC-39-812-s001.docx]

SI

Supporting information (SI) available via the figshare data repository, <https://figshare.com/s/e1da4020bb3083d38ff1> [this name redacted, temporary link allows anonymous viewing by reviewers and will be replaced by a stable doi when published].
